# Identifying Early Warning Signals for the Sudden Transition from Mild to Severe Tobacco Etch Disease by Dynamical Network Biomarkers

**DOI:** 10.3390/v12010016

**Published:** 2019-12-20

**Authors:** Adrián Tarazona, Javier Forment, Santiago F. Elena

**Affiliations:** 1 Instituto de Biología Integrativa de Sistemas (I2SysBio), CSIC-Universitat de València, Paterna, 46980 València, Spain; adriants410@gmail.com; 2 Instituto de Biología Molecular y Celular de Plantas (IBMCP), CSIC-Universitat Politècnica de València, 46022 València, Spain; jforment@ibmcp.upv.es; 3 The Santa Fe Institute, Santa Fe, NM 87501, USA

**Keywords:** complex systems, DNB, phase transitions, plant-virus interaction, potyvirus, protein-protein interaction networks, response to infection, systems biology, tobacco etch virus

## Abstract

Complex systems exhibit critical thresholds at which they transition among alternative phases. Complex systems theory has been applied to analyze disease progression, distinguishing three stages along progression: (i) a normal noninfected state; (ii) a predisease state, in which the host is infected and responds and therapeutic interventions could still be effective; and (iii) an irreversible state, where the system is seriously threatened. The dynamical network biomarker (DNB) theory sought for early warnings of the transition from health to disease. Such DNBs might range from individual genes to complex structures in transcriptional regulatory or protein–protein interaction networks. Here, we revisit transcriptomic data obtained during infection of tobacco plants with tobacco etch potyvirus to identify DNBs signaling the transition from mild/reversible to severe/irreversible disease. We identified genes showing a sudden transition in expression along disease categories. Some of these genes cluster in modules that show the properties of DNBs. These modules contain both genes known to be involved in response to pathogens (e.g., *ADH2*, *CYP19*, *ERF1*, *KAB1*, *LAP1*, *MBF1C*, *MYB58*, *PR1*, or *TPS5*) and other genes not previously related to biotic stress responses (e.g., *ABCI6*, *BBX21*, *NAP1*, *OSM34*, or *ZPN1*).

## 1. Introduction

It has become obvious throughout the years that complex systems all have critical thresholds at which the system suddenly shifts among states [1,2,3]. These classes of events are well known in dynamical systems theory as bifurcations that result in qualitative transitions among states or attractors [4]. Such critical transitions have been described in many different areas of biosciences. For example, in medicine, prostate cancer, asthma attacks, or epileptic seizures are examples of such spontaneous failures. In epidemiology, the triggering of a pandemic out from a locally contained contact network depends on small fluctuations on transmission rates. Ecosystems may collapse after the extinction of some primary producer species, the introduction of foreign ones, or after cataclysmic reductions in biodiversity. These critical thresholds, also known as “tipping points”, are hard to predict because the system shows little change before the tipping point is reached [5]. However, a general dynamical property of such systems, known as “critical slowing down”, may allow for making some educated predictions [6]. Critical slowing down occurs in the vicinity of a bifurcation point where the system becomes increasingly slow in recovering from small perturbations back into its equilibrium state [7]. Increases in variance, temporal autocorrelation, skewness, and spatial correlations are generally expected to occur as a system slows down to a critical point. These statistical properties can be seen as early warning signals [8,9].

**Figure 1:**
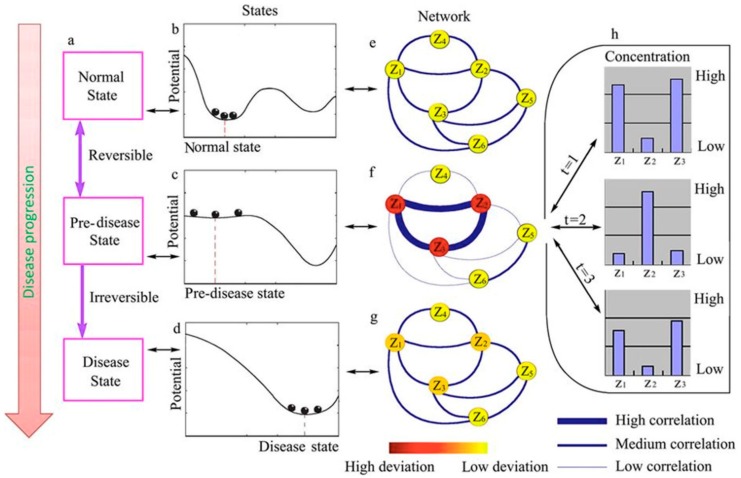
Schematic illustration of the dynamical features of disease progression from a health to a disease state through a predisease state. (**a**–**d**) Representation of the evolution of the dynamical system between the stable equilibria represented by the healthy (b) and disease (d) states via an unstable predisease state (c). (**e**–**g**) Represent a molecular network where the correlations and deviations of different molecules (*z_i_*) are described by the thickness of edges and the color of nodes, respectively. When the system approaches the predisease state, deviations increase drastically, and the correlation among some molecules increases, whereas their correlations with other elements in the network decrease. Molecules *z*_1_, *z*_2_, *z*_3_ represent the dynamical network biomarker (DNB). (h) Examples of the dynamical fluctuations in the concentration of the molecules in the DNB at the pre-disease state. Reproduced under a creative commons license from [10].

Considerable evidence suggests that during the progression of complex diseases (e.g., cancer, autoimmunity, cystic fibrosis, AIDS, flu, etc.) deterioration is not smooth but usually happens in a quite abrupt manner [10]. These critical phenomena generally result in a drastic or a qualitative transition in the underlying molecular networks from a normal state to a disease state, which corresponds to tipping points. The progression of complex diseases are nonlinear processes that can be divided into three stages [11,12]. Rewording these stages to reflect the progression of a viral disease: (i) A normal state, in which the host is not infected and its entire physiology is in a homeostatic state, which is considered to have high resilience and robustness to perturbation due to its dynamical structure (Figure 1). (ii) A predisease state, in which the host has been already infected and responds to infection; it is a latent period that can be seen as the limit of the normal state, which occurs before the imminent phase transition tipping point is reached. In this state, the system is sensitive to the virus perturbations but still reversible to the normal state when it properly controls virus’ replication and spread, but a small change in the parameters of the system may suffice to drive it into collapse through bifurcation, which often implies a critical phase transition to the disease state (Figure 1). (iii) The disease state represents a seriously damaged stage, possibly with high resilience and robustness, where the system usually finds it hard to recover and return to the normal state, even with antiviral interventions, which contrasts with the predisease state (Figure 1). Therefore, it is crucial to detect the predisease state to prevent qualitative deterioration and to further elucidate its molecular mechanisms [13].

For many complex diseases, however, it is a difficult task to predict tipping points at the predisease state because the state of the system may change little from the normal state. Hence, the diagnosis of tipping points by traditional single-gene biomarkers and snapshot static measurements of physiological parameters may not be effective to distinguish between these two states. Another obstacle that hampers the detection of early warning signals is the complexity of viral infectious diseases, which can involve thousands of genetic and epigenetic factors and variation among tissues and organs. A final complication arises from the inherent variation among individuals in their response to infection, even with the same viral isolate. Therefore, detecting the predisease state must be an individual-based predictive endeavor; however, the number of samples available from individuals has been rather limited, unlike many other complex physical systems that are measured over a long-term period with a large number of samples.

This situation is changing due to technological and conceptual advances. Firstly, new high-throughput omic technologies are enabling us to observe gene expression, protein accumulation, and metabolites generation at a genome-wide scale. Such highly dimensional big data not only provide a global overview with rich information on the system of interest, but also represents the accumulated effects of its long-term dynamics [10]. Using the growing amount of available time-course high-throughput omic data obtained during viral infections of individuals and taking advantage of the principle of critical slowing down, it would be possible to identify early warning signals of the transition from the healthy normal state to the irreversible disease state. Secondly, the recently developed dynamical network biomarker (DNB) theory [10,14,15,16,17,18,19,20,21,22,23,24,25,26,27,28,29], may help in identifying early warning signals indicating an imminent phase transition towards disease. Such DNBs should range from individual molecules (genes, proteins, metabolites) or more complex structures (subnetworks, modules, or pathways) in protein–protein interaction networks (PPINs), transcriptional regulatory networks (TRNs), metabolic networks, or noncoding RNA-mediated regulation of DNA epigenetic marks that are leading towards the critical transitions. For illustrative purposes, the small network depicted in Figure 1e–g represents a hypothetical DNB. In short, this small DNB is characterized by showing an increased correlation among its component elements (in this example, the subnetwork formed by molecules *z*_1_, *z*_2,_ and *z*_3_) and a decreased correlation with other components of the network (in this example, molecules *z*_4_, *z*_5,_ and *z*_6_). The graphs in Figure 1h illustrate the fluctuations of the molecular concentrations for the DNB in the predisease state, which dynamically change with strong temporal deviations but are strongly correlated.

In this study, we have revisited the transcriptomic data generated by Cervera et al. [30] from tobacco plants infected with seven different genotypes of tobacco etch potyvirus (TEV). Infected plants reached a more or less advanced disease state depending upon the TEV genotype inoculated, ranging from asymptomatic or disperse chlorotic spots to mild etching and to systemic intense necrotic etching and plant dwarfism. Disease statuses were associated with an increase in the magnitude of the perturbation suffered by the plant transcriptome. The aim of this study was to evaluate the usefulness of the DNB theory to identify possible genes or network elements that may be used as early warning signals of the transition from an asymptomatic or mild disease to a severe one. To this end, we have first identified genes showing a significant biphasic behavior across disease states. These genes have been mapped into PPIN and TRN and the statistical properties of the modules evaluated against the predictions of the DNB theory. We found groups of genes that consistently showed the dynamical properties of a DNB. We found that the phase transition between the reversible predisease state and the severe irreversible disease took place nearby the disease state induced by a particular mutant genotype. The DNB was formed both by genes previously described as related to response to pathogens as well other genes not previously associated to disease. Coordinated alterations in the expression of these genes could serve as early warning for the irreversible transition to a sever disease syndrome.

## 2. Materials and Methods

### 2.1. Description of the Nicotiana tabacum—TEV Pathosystem and Transcriptomic Data

**Figure 2:**
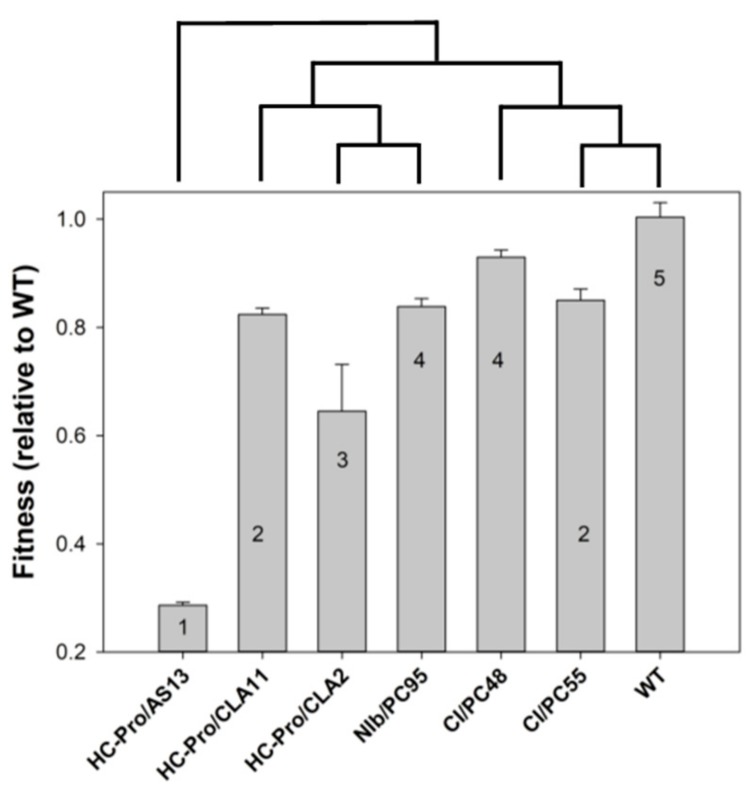
Relationship between the relative fitness of each tobacco etch potyvirus (TEV) genotype (ordinate), the severity of symptoms they induce in *N. tabacum* plants (numbers inside the bars, semiquantitative scale ranging from 1 to 5), and the similarity in the transcriptomic profile of infected plants (dendrogram in top). Error bars represent ±1 SEM.

Cervera et al. [30] infected *Nicotiana tabacum* L. cv. Xanthi *NN* plants with seven genotypes of TEV (genus *Potyvirus*, family *Potyviridae*). Six of these genotypes were previously generated by site-directed mutagenesis on an infectious clone obtained from a wild-type (WT) isolate from tobacco (GenBank accession DQ986288) and they only differed from the WT genotype in the mutagenized nucleotide sites [31,32]. Six of these TEV genotypes induced symptoms of increasing severity eight days post-inoculation (dpi); HC-Pro/AS13 showed a retard in symptoms development, being visible 15 dpi: HC-Pro/AS13 (amino acid substitutions E299A and D300A) induced disperse chlorotic spots, with the newest leaves showing no symptoms at all; HC-Pro/CLA11 and CI/PC55 (amino acid substitutions E443K and K494N in the respective proteins) showed mild etching symptoms; HC-Pro/CLA2 (substitution V192A) showed more intense chlorotic etching but without necrosis; NIb/PC95 and CI/PC48 (amino acid substitutions A473S and H156Q in the respective proteins) showed intense etching in all new leaves and plant dwarfism, though less severe than the necrotic etching and dwarfism induced by the WT virus (see Figure 1 in [30]). Figure 2 summarizes all the relevant information defining the different disease states. Figure 2 also shows a significant positive association between viral fitness and the severity of symptoms induced by each TEV genotype (Spearman’s *r_S_* = 0.7638, 5 d.f., *p* = 0.0384). More interestingly, the magnitude of the fitness difference between each mutant and the WT was shown to be a good predictor of the size and direction of the perturbation induced by infection in the plant transcriptome (see Figures 2B and 3B in [30]). The dendogram in top of Figure 2 shows the clustering of TEV genotypes according their similarity in the list of differentially expressed genes (DEGs). It strongly suggests that early disease states (HC-Pro/AS13), characterized by mild symptoms 15 dpi and very few altered DEGs compared to healthy plants (see Figure and 3B in [30]), showed very dissimilar transcriptomic responses in comparison to plants in the most advanced disease state 8 dpi and with very severe symptoms (WT). As the etching disease state became more severe at 8 dpi, so did the number of altered DEGs, which overlapped more with the list of altered DEGs in plants infected with WT (see Figure and 3B in [30]).

Pooling together all these pieces of information, we have classified the progression of etch disease into seven categories 8 dpi, I to VII, each associated to infection with a particular genotype of TEV. Disease category I corresponds to the infection with TEV genotype HC-Pro/AS13, category II to infection with genotype HC-Pro/CLA2, category III to plants infected with genotype HC-Pro/CLA11, category IV to infection with genotype NIb/PC95, and the most severe categories V–VII correspond to the infection of plants with genotypes CI/PC55, CI/PC48, and WT, respectively. Hereafter, we will refer to the disease categories.

The methods employed to generate these transcriptomic data are provided in [30]. The raw microarray data were downloaded from NCBI GEO under accession number GSE99838. Individual probes in the Agilent’s tobacco microarray were annotated and updated against the latest version of the *N. tabacum* mRNA database available at the Sol Genomics Network [33] following the procedure described in [30]. A list of 40,430 annotated probes was generated, with 2673 probes pointing to alternative splicing forms of the same genes.

### 2.2. Identification of Genes with Biphasic Gene Expression Pattern along Disease Categories

As a first step, we tested which of the 40,430 probes showed a biphasic pattern of expression along the different etch disease categories. The significance of biphasity was statistically tested for each gene using the one-sample runs test with the mean log_2_-fold change value across disease categories as cutting point (runs.test function in R version 3.6.1) and correcting for multiple comparisons using the Benjamini and Hochberg false discovery rate (FDR) method [34]. Plant genes showing random fluctuations in expression across the seven disease categories were not further considered. Genes that rejected the null hypothesis of random fluctuations, i.e., showed monotonous increases or decreases of expression across the disease categories, or genes that showed biphasic changes in expression level (e.g., low expression for early disease categories and a sudden increase in expression for plants at advanced disease categories) were retained for further analysis.

### 2.3. Criteria for Identification and Statistical Evaluation of DNB

DNBs were identified using the method originally proposed by Chen et al. [10] and later on improved by Liu et al. [19,22] to account for noisy data or to analyze single health vs. disease samples [12]. These authors derived network-based dynamical criteria to identify genes, or groups of genes, as early warning signals indicating the immediate bifurcation before a critical transition to disease occurs. The following three properties hold when the system reaches the predisease state: (i) A dominant group of genes exist, whose expression levels change in a coordinated manner. Statistically speaking, the average Pearson correlation coefficient among the expression of all pairs of genes in the dominant group (〈*r_d_*〉) drastically increases in absolute value. (ii) Simultaneously, a decoupling exists between the expression level of these genes and any other gene outside the dominant group. Statistically speaking, this is equivalent to saying that the average Pearson correlation coefficient between elements in the dominant group and outside (〈*r_o_*〉) should decrease in absolute value. Finally, (iii) the average standard deviations (〈*σ_d_*〉) of molecules in the dominant group drastically increase. If these three conditions are simultaneously satisfied, then the dominant group is a DNB that reflects the transition of the system to the irreversible disease state. To obtain a stronger signal of the predisease state, Chen et al. [10] proposed combining these three criteria into the following composite index: *I* = 〈*σ_d_*〉|〈*r_d_*〉|/|〈*r_o_*〉|, where |·| represents the absolute value of the corresponding average correlation coefficients. Normalized *I* values, I*, were generated by dividing the *I* values obtained for a dominant group across all disease categories (DC) by the minimum observed I value: $I^{*}=I/\underset{k\subset DC}{\min}\left(I_{k}\right)$.

A Python script, DNB.py, was written that incorporates both the filtering algorithm for identifying biphasic genes and computes the I* values (available from GitHub at https://github.com/AdriTara/DNB).

### 2.4. Functional Enrichment Analysis of DNBs

The list of *N. tabacum* orthologous genes in the *Arabidopsis thaliana* genome was generated using BLAST against TAIR version 10 database of *A. thaliana* cDNAs [35] with a cutoff *e*-value <10^−4^ [30]. The determination of the gene ontology (GO) categories overrepresented within the lists of DNBs was done in the PANTHER webserver [36] by using the Fisher’s exact test (with FDR adjusted to *p* < 0.05 according to Benhamini and Yekutieli criterion [37]).

### 2.5. Network Analyses

Cytoscape version 3.7.2 [38] was used for three main purposes. First, for mapping the biphasic genes into the *A. thaliana* PPIN version AI-1 (downloaded from: interatome.dfci.harvard.edu/A_thaliana/) [39] and into the *A. thaliana* TRN inferred by Carrera et al. [40]. Second, for calculating the statistical properties of the DNB subnetworks and of the entire PPIN and TRN (clustering coefficients, degree distributions, shortest paths, neighborhood connectivity, eccentricity, topological coefficient, and critical exponent of the degree distribution). Finally, it was used for visualization of the DNBs in the context of the global networks.

## 3. Results and Discussion

### 3.1. Distribution and Characterization of Genes Showing Biphasic Expression Profiles across Disease Categories

**Figure 3:**
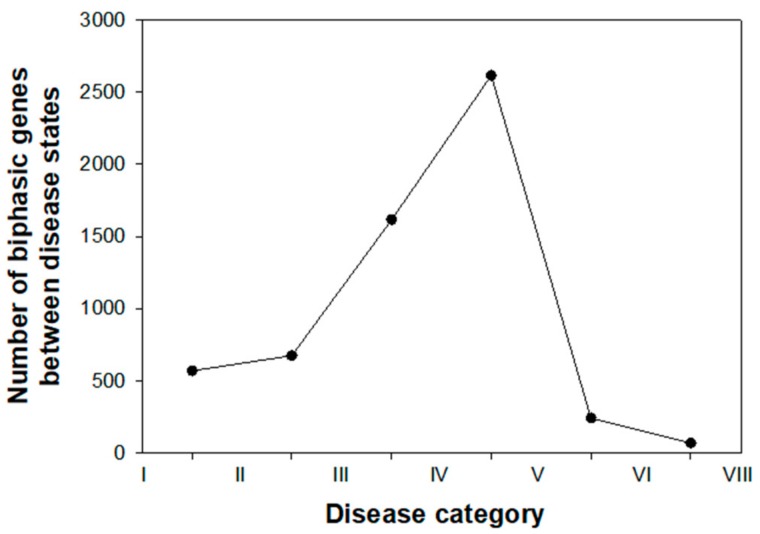
Number of significant sudden phase transitions observed in the levels of gene expression (log_2_-fold change) along the seven disease categories defined in Section 2.1.

In our quest for putative DNBs, our first step was to study the number of genes showing a significant shift in expression (log_2_-fold change) relative to control mock-inoculated plants. Figure 3 illustrates the results from this study. The number of genes experiencing a sudden change in expression profile at the two earliest states of disease progression were ~500. However, a dramatic increase in the number of genes experiencing such sudden transition in expression happened in the next two disease categories, with a maximum value of 2,616 reached between the disease categories IV and V. Thereafter, the expression of very few genes showing a significant biphasic expression pattern can be interpreted as first evidence that genes relevant to predict the progression of disease from reversible to irreversible states would have been already affected by infection at the disease category IV.

From the previous paragraph, we can conclude that genes can be classified into three groups according to the disease category at which they experience a sharp phase transition in their expression: early (transitions between disease categories I–III), intermediate (between disease categories III–V), and late (between disease categories V–VII). This classification is further supported by transcriptomic data: the peak observed in Figure 3 coincides with the split in the two main clusters in gene expression patterns shown in top of Figure 2—disease categories II–IV showing a smaller perturbation in the plant transcriptome than observed in plants at the more advanced disease categories V–VII (see also Figure 3A,B in [30]).

Next, we sought to characterize the genes showing a significant biphasic pattern at each one of the disease categories. To do so, we ranked genes from largest to smallest change in the absolute value of the log_2_-fold change expression between consecutive disease categories (Supplementary Table S1). Functional annotation was performed from the gene lists of each group (Supplementary Table S2). As illustrative examples, here we will just mention the three most extreme cases observed for the transition between disease categories and a few representative cases of enriched functional GO terms. Notice that only cases in which an orthologous in *Arabidopsis* was found for the tobacco gene were included in these studies.

#### 3.1.1. Characterization of Early Biphasic Genes

First, the three top early genes that exhibit a significant phase transition between disease categories I and II are At3g23240 (*ETHYLENE RESPONSE FACTOR 1*, *ERF1*), At2g44010 (hypothetical protein), and At4g11650 (*OSMOTIN 34*, *OSM34*), with all three cases with positive changes in expression. *ERF1* transcription factor belongs to the *APETALA2*/*ERF* family and is a well-known regulator of molecular responses against pathogens attacks [41]. ERFs also bind to dehydration-responsive elements and act as hub coordinators in plant responses to abiotic stresses [41]. *OSM34* also encodes for a protein involved in wide responses to stresses. It has been described as a stress-responsive cytotoxic antifungal protein belonging to the pathogenesis-related (PR)-5 family that confers tolerance both to biotic and abiotic stresses [42]. It is worth mentioning that among the top 5% of the biphasic genes listed in this disease category, two more PR proteins were included: *PR1* (At2g14580) and *PR4* (At3g12500). Functional annotation of all genes in this group found significant enrichment in only two GO terms: response to stress (GO:0006950) and multiorganism process (GO:0051704).

Second, looking at the top early three genes with a significant sudden transition in expression between disease categories II and III, we observed significant decreases in the expression of At3g20440 (*EMBRYO DEFECTIVE 2729*, *EMB2729*) and At5g15680 (armadillo (ARM)-repeat superfamily protein), and a significant increase in the expression of At5g26800 (Xaa-Pro aminopeptidase P). The protein encoded by *EMB2729* belongs to the α-amylase family, has plastidic localization, and is involved in the catabolism of starch to yield glucose and maltose. Starch is well-known for its role in several abiotic stresses (e.g., extreme temperatures and drought) [43], but recently another amylase, *AMY1*, has been described in response to infection of *A. thaliana* with *Pseudomonas syringae* [44]. ARM repeat-containing proteins are very common in *A. thaliana* and most of them contain a U-box E3 ubiquitin ligase domain [45]. Ubiquitinoylation plays an essential role in hormone synthesis, hormonal signaling cascades, and plant defense mechanisms [46]. Hence, a reduction in the expression of this gene may result in lower levels of ubiquitination of proteins involved in, for example, abscisic acid (ABA) and jasmonic acid (JA) signaling pathways, and thus directly impact the response to infection. The role of Xaa-Pro aminopeptidase P in biotic stress in plants has not been studied. However, cleavage of the Xaa-Pro motif may initiate the proteolytic degradation and inactivation of many small peptides. The role of such small peptides as signaling molecules in the regulation of plant defense, growth, and development has been anticipated [47]. Functional annotation of all genes in this group found significant enrichment in 20 GO terms. Among them, response to bacterium (GO:0009617), cellular response to oxygen-containing compound (GO:1901701), regulation of photosynthesis (GO:0010109), response to abiotic stimulus (GO:0009628), response to stress (GO:0006950), and metabolic process (GO:0008152).

In conclusion, genes experiencing a sharp transition in gene expression during early disease categories were mainly involved in pathogen detection and in dysregulation of photosynthesis.

#### 3.1.2. Characterization of Intermediate Biphasic Genes

Third, the top three intermediate genes in the transition between disease categories III and IV were At5g11810 (rhomboid family protease), At5g60020 (*LACCASE 17*, *LAC17*), and At5g48460 (actin-binding calponin homology (CH) domain-containing protein). The three genes showed a downshift in expression during this transition. Rhomboid-like proteases are intermembrane serine proteases conserved across all kingdoms. They participate in functions as diverse as growth factor signaling, mitochondrial dynamics, parasite evasion, and the machinery of protein quality control [48]. LAC17 protein is involved in lignin metabolism and detoxification of lignin-derived products and is essential for vascular development in *A. thaliana*. *LAC17* downregulation by infection would explain the dwarfism observed in plants infected with these viral genotypes. Functional annotation of all genes in this group found significant enrichment in 92 GO terms. Here, we just mention leaf senescence (GO:0010150), plant organ senescence (GO:0090693), positive regulation of autophagy (GO:0010508), photosynthesis, light harvesting in photosystem I (GO:0009768), α-amino acid catabolic process (GO:1901606) nucleic acid metabolic process (GO:0090304), RNA metabolic process (GO:0016070), RNA processing (GO:0006396), rRNA metabolic process (GO:0016072), ncRNA processing (GO:0034470), RNA splicing (GO:0008380), cellular component organization or biogenesis (GO:0071840), and plant organ development (GO:0099402).

Fourth, focusing now on the transition between disease categories IV and V, in which the largest number of genes with biphasic transition was observed, the top three intermediate genes with the largest changes in gene expression were At4g27030 (*FATTY ACID DESATURASE A*, *FADA*), At3g55580 (regulator of chromosome condensation (RCC1) family protein), and At5g16010 (3-oxo-5-α-steroid 4-dehydrogenase family protein). *FADA* expression was actually upregulated, while the other two genes were down expressed. *FADA* is a chloroplast membrane-associated protein with Δ3-trans-hexadecenoic acid phosphatidylglycerol desaturase activity and also ubiquitin ligase and binding activities. Fatty acid mobilization and regulation is important during plant response to stress [49]; adjusting membrane fluidity keeps the conditions for integral proteins to operate under different stressful conditions. For instance, chloroplast oleic acid levels are critical for normal pathogen defense responses, which include programmed cell death and systemic acquired resistance, and fatty acid-related signals modulate the crosstalk between different defense signaling pathways [49]. *RCC1* is a master regulator of multiple cellular processes. It is important for nucleo-cytoplasm trafficking, mitosis, nuclear membrane assembly, control of chromatin agglutination during S phase of mitosis, assembly of mitochondrial oxidative phosphorylation systems, and resistance to abiotic stresses (e.g., UVB radiation, salinity and cold tolerance) [50]. The 3-oxo-5-α-steroid 4-dehydrogenase protein is associated with the chloroplast envelope and its expression has been observed to be affected by geminivirus infection [51]. Functional annotation of all genes in this group found significant enrichment in 200 GO terms, worth mentioning here: component organization or biogenesis, cellular component organization (GO:0016043), organelle organization (GO:0006996), cytoplasmic mRNA processing body assembly (GO:0033962), protein import into chloroplast stroma (GO:0045037), protein export from nucleus (GO:0006611), RNA localization (GO:0006405), RNA export from nucleus (GO:0006406), cellular response to unfolded protein (GO:0034620), defense response to other organism (GO:0098542), multiorganism process, and reproductive structure development (GO:0048608).

In conclusion, genes experiencing a phase transition in their expression at intermediate disease categories were involved in response to infection, plant senescence and developmental defects, RNA and protein processing and trafficking, and reorganization of cellular components, especially organelles.

#### 3.1.3. Characterization of Late Biphasic Genes

Fifth, among the few late genes that showed a sharp transition in expression between disease categories V and VI, we would highlight the significant increase in the expression of At2g32850 (protein kinase superfamily protein) and the decrease in the expression of At3g54480 (*SKP1/ASK-INTERACTING PROTEIN 5*, *SKIP5*) and At2g32400 (*GLUTAMATE RECEPTOR 5*, *GLR5*). Protein Ser/Thr kinases represent about 4% of *A. thaliana* proteome and they play essential roles in sensing, mediating, and coordinating cellular responses to a very large array of stimuli [52]. Thus, overexpression of protein kinase genes may just result from the overstimulation induced by viral infection. *SKIP5* contains F-box and cyclin-like domains. It is part of the *Arabidopsis* SKP1-related proteins (ASK) complex that regulates polyubiquitination and protein degradation [53]. *GLR5* is involved in response to stress by mediating the homeostasis of cellular Ca^2+^ [54]. Endoplasmic reticulum Ca^2+^ signaling is implicated in a large number of coordinated cellular processes and is tightly regulated, and it shows crosstalk with nitric oxide, ROS, and MAPKs signaling pathways that ultimately lead to plant tolerance [55]. Downregulation of *GLR5* would result in a loss of Ca^2+^ homeostasis at advanced states of the disease. Functional annotation of all genes in this group found significant enrichment in only one very general GO term, cellular process (GO:0009987).

Finally, the last set of genes showing a sharp transition was between advanced disease categories VI and VII. Significant increase in the expression of At1g75170 (Sec14p-like phosphatidylinositol transfer family protein), At4g18240 (*STARCH SYNTHASE 4*, *SS4*), and At4g23160 (*CYSTEINE-RICH RECEPTOR-LIKE KINASE*, *CRK8*) were observed. Phosphatidylinositol transfer proteins (PITPs) are widely distributed across kingdoms and in plants are also known as patellin (PATL) proteins. PATLs play roles in plant development and stress tolerance [56]. For instance, altered expression of several PATL genes was already observed in *A. thaliana* plants infected with geminivirus [51]. PATL are regulators in diverse signaling pathways, for instance in auxin signaling, the salt overlay sensitive (SOS) pathway, calmodulin-mediated sensing, and deubiquitinization [56]. *A. thaliana PATL3* and *PATL6* have been shown to interact with alfalfa mosaic alfamovirus movement protein, suggesting that PATL proteins are involved in viral cell-to-cell movement [57]. *SS4* is involved in the metabolism of starch. Overexpressing *SS4* goes in parallel with the underexpression of the α-amylase EMB2729 discussed above, and the final goal may be an attempt of the cell to maintain starch concentration. Receptor-like protein kinases contain Cys-rich repeats in their extracellular domains. Genes encoding many CRKs are induced by pathogen infection; in particular, *CRK8* has already been shown to participate in activation of hypersensitive cell death [58], and its expression was also altered upon geminivirus infection [51]. Functional annotation of all genes in this group found no significant enrichment in GO terms.

### 3.2. Mapping Biphasic Genes into A. thaliana AI-1 PPIN

**Figure 4:**
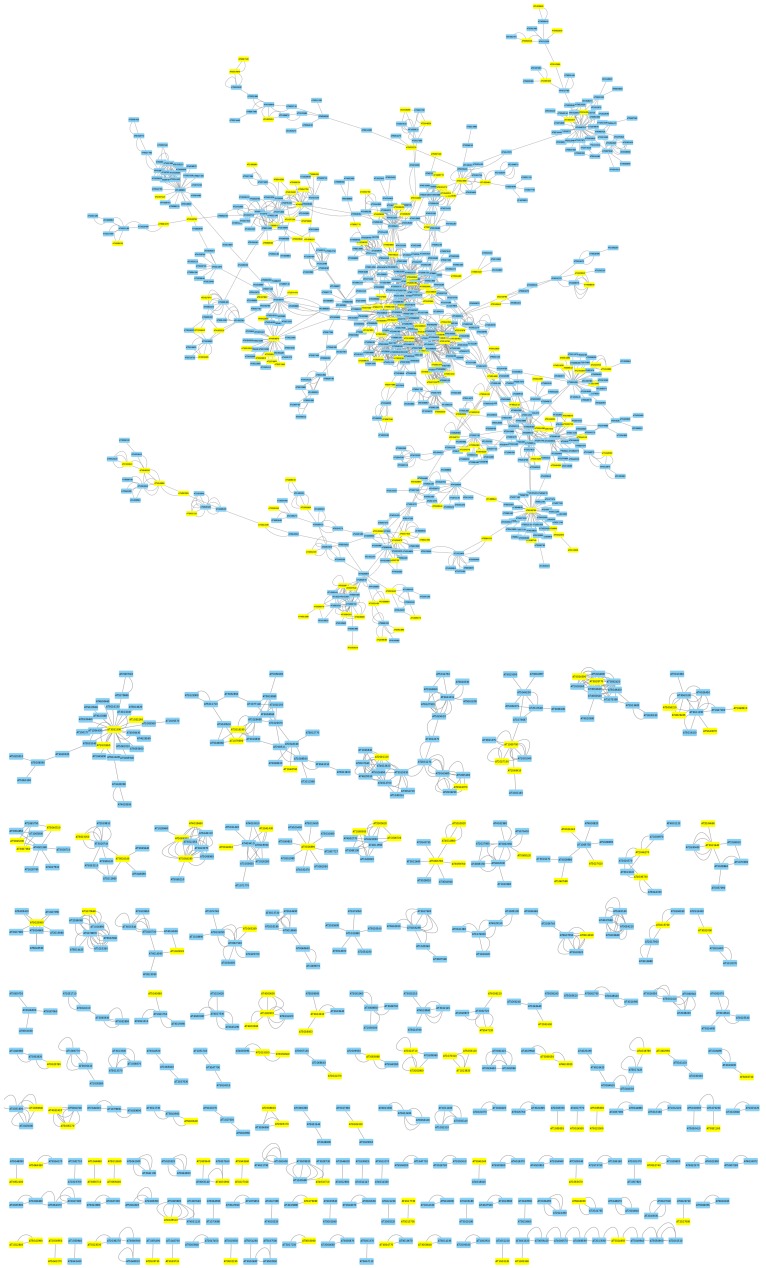
Mapping of all genes showing a biphasic pattern (in yellow) in their expression into the *A. thaliana* AI-1 protein–protein interaction networks (PPIN).

Genes with biphasic expression patterns along disease categories identified in the previous section were mapped into the *A. thaliana* AI-1 PPIN (Figure 4). A total of 121 modules contain at least one of the biphasic genes (minimum 1, maximum 119) (Supplementary File S1). The median number of biphasic genes per module was 3 (interquartile range 3), though this figure obviously depends on the size of the module, as larger modules are expected to contain more biphasic genes than smaller ones.

#### Inference of PPIN-Based DNBs

**Figure 5:**
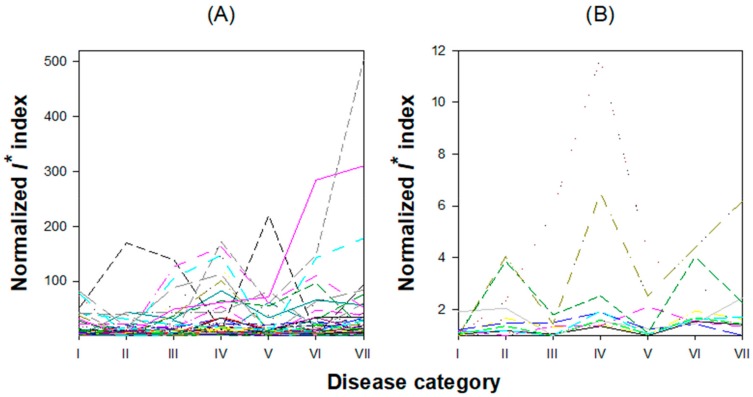
Evolution of the I* index along disease categories. (**A**) For each of the 121 candidate PPIN-based DNBs. (**B**) For each of the 11 candidate transcriptional regulatory network (TRN)-based DNBs. In both cases the largest number of peaks in I* corresponds to the disease category IV.

Now, for each one of these 121 modules, we applied the algorithm described in Section 2.3 to determine whether any of them fulfil the three necessary mathematical conditions proposed by Chen et al. [10] to be considered as a DNB. At each disease category, we calculated the I* index for each of the 121 modules. Figure 5A shows the evolution of I* along disease categories (correlation coefficients, standard deviations, and I* values are all reported in Supplementary File S1). The I* index shows a great amount of variability among modules, as well as among disease categories, with some modules showing no clear peaks and others showing peaks at different disease categories. However, disease category IV shows the largest number of dominant groups for which the I* index takes a maximum value; the most advanced disease categories (VI and VII) also show a considerable amount of I* peaks (Figure 5A). To further filter out noise and reduce the number of potential candidates for ^PPIN^DNBs to the most significative cases, we set up a minimum I* threshold value at 〈I*〉 + 2σ_I*_ = 11.8201 + 2 × 4.6926 = 21.2052. The number of modules fulfilling this more stringent criterion was 45. To some extent unexpected, these dominant groups contained few genes: at the one side, 27 of these modules contained only two genes and, at the other side, the largest module contained only nine genes. Hereafter, we will refer to them as ^PPIN^DNB candidates. Supplementary File S1 also contains the I* values calculated for each module across disease categories and the corresponding lists of genes per module. For the sake of illustration, here we will just comment the two ^PPIN^DNB candidates with the largest I* at disease category IV: ^PPIN^DNBs 134 and 175. ^PPIN^DNB-134 is composed by only two genes, At3g24500 (*MULTIPROTEIN BRIDGING FACTOR 1C*, *MBF1C*) and At4g17770 (*TREHALOSE PHOSPHATE SYNTASE 5*, *TPS5*). *MBF1C* is a highly conserved transcriptional coactivator that may serve as a bridge between a bZIP factor and a TATA-binding proteins (TBP). Its expression is especially high in response to pathogen infection and other abiotic stresses and after exogenous treatment with ABA and salicylic acid (SA). *MBF1C* is also supposed to regulate the activation of genes encoding heat shock proteins (HSP) [59]. Pathogen infection increases trehalose levels and upregulates *TPS5* expression [60]. Elevated trehalose production results in increased susceptibility to biotrophic pathogens but reduced disease severity in necrotrophic ones. Expression of *TPS5* has also been related with a suppression of *PR1,* and thus with enhanced susceptibility to infection [60]

^PPIN^DNB-175 is composed by genes At4g04770 (*NUCLEOSOME ASSEMBLY PROTEIN 1*, *NAP1*) and At3g10670 (*ABC TRANSPORTER I FAMILY MEMBER 6*, *ABCI6*). Both proteins belong to the ATP-binding cassette (ABC) superfamily [61]. *NAP1* acts as histone chaperone functioning in cell proliferation. NAP1 interacts with NUCLEOSTEMIN-LIKE 1 (NSN1) to coordinate cell proliferation [62]; an incorrect interaction, or a lack of, may explain some of the growth deformations observed in advanced disease categories. ABCI6 (also known as NAP7) is a transmembrane ATP chloroplastic transporter, previously not associated to biotic stresses.

Given the small number of genes contained in the 45 candidate ^PPIN^DNBs, no candidate significantly enriched in functional GO terms was found.

### 3.3. Mapping Biphasic Genes into A. thaliana TRN

As mentioned above, Figure 4 illustrates the sparse topology of AI-1 PPIN, with a single large connected component and a number of small components, many of which only contain two or three nodes. As a consequence, our ability to detect large DNBs was constrained by these topological properties of the PPIN. To explore whether a more connected network will result in an increased number of candidate DNBs, we repeated the same analyses as in Section 3.2, but now using the TRN model described in [40]. This TRN model is characterized by a very large and densely connected component, with significant modularity and small-world properties, plus a few smaller disconnected components [40].

#### Inference of TRN-Based DNBs

Genes with biphasic patterns of gene expression along disease categories were mapped into the TRN model (Supplementary File S2). A total of 19 dominant groups contained at least one of these biphasic genes, all potential candidates for ^TRN^DNBs. The median number of biphasic genes per candidate ^TRN^DNB was 42.5 (interquartile range 128.5), ranging from the 18,980 genes in ^TRN^DNB-1 to the three genes that define ^TRN^DNB-34. The largest I* values were found for the disease category IV (Supplementary File S2). Again, to further filter out noise and reduce the number of potential candidates for ^TRN^DNBs to the most significative cases, we set up a minimum I* threshold value at 〈I*〉 + 2σ_I*_ = 1.9635 + 2 × 0.5948 = 3.1531. Only ^TRN^DNBs 34, 36, and 40 resisted this more stringent criterium. ^TRN^DNB-34 shows its maximum I* at the disease category IV. It contains three biphasic genes: At1g04290, At1g75540 (*B-BOX DOMAIN PROTEIN 2*1 (*BBX21*), *SALT TOLERANCE HOMOLOG 2* (*STH2*) and *LONG HYPOCOTYL UNDER SHADE* (*LHUS*)), and At3g02830 (*ZINC FINGER PROTEIN 1* (*ZFN1*) and *PENTA 1* (*PNT1*)). At1g04290 encodes for a member of the thioesterase superfamily protein located in the peroxisome and with acyl-CoA hydrolase activity. No information exists about a specific role of thioesterases in the defense against pathogens. However, mounting evidence assigns defensive roles to leaf peroxisomes [63]. *BBX21* is a B-box Zn finger transcription factor that interacts, for instance, with *CONSTITUTIVE PHOTOMORPHOGENIC 1* (*COP1*) to control de-etiolation, shade avoidance, and stomatal movement. Alteration of *BBX21* expression affects the rate of photosynthesis [64]. *ZFN1* is a negative regulator of translation. *ZFN1* has been shown to be a regulator of the plant responses to light via its cytoplasmic interaction with phytochromes and inhibition of the *PROTOCHLOROPHYLLIDE REDUCTASE* (*PORA*) mRNA [65]. The coordinated deregulation of *BBX21* and *ZFN1* may contribute to explain why at advanced disease categories plants show chlorosis and yellowing. Therefore, ^TRN^DNB-34 is defined by alterations in photosynthesis.

**Figure 6:**
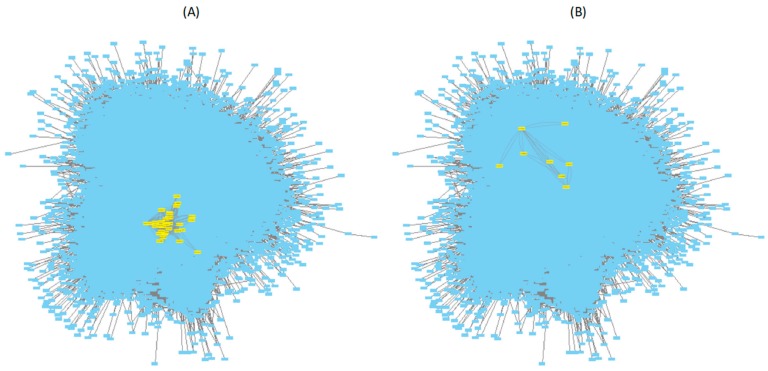
Mapping of genes belonging to (**A**) ^TRN^DNB-36 and (**B**) ^TRN^DNB-40 (in yellow) into the *A. thaliana* TRN model [40].

^TRN^DNB-36 contains 40 genes (Figure 6A), with the maximum I* at the disease category VI (see Supplementary File S2 for the list of genes). The three most significant biphasic genes belonging to this DNB are At1g02720 (*GALACTURONOSYLTRANSFERASE-LIKE 5*, *GATL5*), At1g04380 (encodes a protein similar to a 2-oxoglutarate-dependent dioxygenase), and At1g16490 (*MYB DOMAIN PROTEIN 58*, *MYB58*). *GATL5* is involved in pectin synthesis in seed mucilage but, so far, has not been related to responses to biotic stresses. 2-oxoglutarate-dependent dioxygenases are involved in the biosynthesis of glucosinolates, secondary metabolites that act as precursors of chemical defenses against herbivores [66]. *MYB58* is a member of the R2R3 transcription factor family. R2R3-MYB factors are hubs in regulatory networks controlling development, metabolism, and responses to biotic and abiotic stresses [67]. All 40 genes in ^TRN^DNB-36 are significantly enriched in a variety of functional GO terms: glycoprotein fucosylation (GO:0036071), fatty acid homeostasis (GO:0055089), somatic embryogenesis (GO:0010262), seed development (GO:0048316), regulation of secondary metabolic processes (GO:0043455), positive regulation of macromolecule biosynthesis process (GO:0010557), regulation of gene-specific transcription and transcriptional control (GO:0006355), and regulation of gene expression (GO:0010468). All these GO terms are, in one way or another, related to secondary metabolism, control of seed development, and regulation of gene expression.

^TRN^DNB-40 contains eight genes with, once again, the maximum I* at the disease category IV (Figure 6B). These genes are At1g04690 (*POTASSIUM CHANEL BETA SUBUNIT 1*, *KAB1*), At1g56450 (*20S PROTEASOME BETA SUBUNIT G1*, *PBG1*), At2g16600 (*CYCLOPHILIN 19*, *CYP19*), At2g24200 (*LEUCYL AMINOPEPTIDASE 1*, *LAP1*), At3g02630 (*ACYL ACYL CARRIER PROTEIN (ACP) DESATURASE 5*, *AAD5*), At4g26130 (hypothetical protein described as a cotton fiber protein), At5g2107 (hypothetical protein described as a Fe^3+^ di-citrate transport system permease), and At5g43940 (*ALCOHOL DEHYDROGENASE 2* (*ADH2*), *PARAQUAT RESISTANT 2* (*PAR2*), and *SENSITIVE TO HOT TEMPERATURE 5* (*HOT5*)). *KAB1* regulates K ion transmembrane transport. The critical role of K homeostasis in plant response to biotic and abiotic stresses [68] is well known. It was shown some years ago that potyvirus HC-Pro targets the 20S proteasome, inhibiting its endonuclease activity [69], thus it is not surprising that plants tend to compensate for this failure by increasing the expression of *PBG1*. *CYP19* belongs to the conserved family of plant immunophilins. Knock-out of *CYP19* in *A. thaliana* results in increased susceptibility to infection with *P. syringae*, whereas overexpressing it results in the alteration of the transcriptomic profile of pathogen-related defense genes and leads to enhanced resistance [70]. *CYP19* is also involved in the production of ROS species [70]. *LAP1* is a moonlighting protein that can act as a chaperone to alleviate stress-induced damages [71]. *HOT5* regulates SA signaling and thermotolerance by modulating the intracellular level of S-nitrosothiol. *PAR2* acts downstream of superoxide to regulate cell death through modulation of intracellular NO concentration [72]. Thus, some of the genes in ^TRN^DNB-40 are involved in different aspects of plant response to stress. No significant enrichment in GO terms was found for the genes belonging to ^TRN^DNB-40.

### 3.4. Topological Properties of the DNBs Subnetworks

It has been previously shown that the host’s DEGs upon infection do not represent a random sample of the host genome but are significantly enriched in highly connected hub genes [73,74]. Usually hub genes are master regulators of downstream processes, a situation that allows viral proteins to take over cellular control in a highly efficient manner [75]. The question that pops up is whether genes belonging to DNBs are also more central and highly connected than a set of genes randomly taken from the entire network. To test this possibility, we estimated several quantitative measures of network topology. Table 1 summarizes these physical properties for the ^PPIN^DNBs and ^TRN^DNBs in comparison to the corresponding values generated for the entire PPIN and TRN. In the case of the ^PPIN^DNBs, the shortest path and neighborhood connectivity show significant differences; alas they point towards the opposite direction from our expectation. On average, the shortest path was 3.43% (*p* = 0.0095) longer for the ^PPIN^DNB subnetworks than for the entire PPIN and the neighborhood was 41.37% (*p* < 0.0001) less connected for the ^PPIN^DNB subnetworks than for the entire PPIN. However, these differences in parameters might just result from the sparseness of the PPIN (Figure 4).

In the case of the ^TRN^DNB subnetworks, all topological parameters, except the betweenness centrality, were significantly different between the ^TRN^DNB subnetworks (not including ^TRN^DNB-1, which was removed from the analyses given its very large size) and the entire TRN (Table 1). On average, the shortest path was 3.48% (*p* < 0.0001) shorter for the ^TRN^DNBs subnetworks than for the entire TRN. The shortest path measures the shortest physical distance between any pair of nodes. Differences in closeness centrality show that, on average, ^TRN^DNBs subnetworks were 3.46% (*p* = 0.0061) closer among them than expected from the entire TRN. Closeness centrality is related to shortest path: the more central a node, the closer it is to all other nodes. More interestingly, average degree, namely the number of connections of a given node, was 53.65% (*p* < 0.0001) larger for the nodes in the ^TRN^DNBs subnetworks, suggesting that they were much more connected than for random sets of genes taken from the TRN. In agreement, the eccentricity was 95.08% (*p* < 0.0001) smaller for the ^TRN^DNBs subnetworks than for the entire network. A lower eccentricity means that the distance between any pair of nodes in the ^TRN^DNBs subnetworks was shorter than for any random pair of nodes in the entire network. Eccentricity is used as a measure of the importance of a node within a network, thus this difference also points towards the potential role as hubs of the ^TRN^DNBs elements. Likewise, on average, the neighboring nodes were also 67.40% (*p* < 0.0001) more connected than expected for the entire TRN. Neighborhood connectivity is the average connectivity of the neighbors of a given node. The two later measures also suggest the existence of positive assortativity; that is, highly connected elements in the ^TRN^DNBs subnetworks tend to be connected with nodes that are also highly connected. In conclusion, with the exception of the clustering and topological coefficients, which were significant yet in the unexpected direction, all centrality measures shown in Table 1 support the idea that genes belonging to the ^TRN^DNBs are very central and highly connected modules in the architecture of the TRN network.

As a final test for an enrichment in highly connected elements in the DNBs, we compared the critical exponents of the power law fitted to the degree distributions of the DNB subnetworks and to the entire networks, as proposed in [73,74]. If DNB subnetworks are enriched in highly connected elements, then we expect the power law degree distribution to be flatter than the one observed for the entire network in a log-log scale. Vice versa, if the DNB subnetworks are enriched in poorly connected elements, then we expect the power law degree distribution to be steeper than the one observed for the entire network in log-log scale. If the DNB subnetworks are a random sample from the entire network, then no differences in critical exponents should be expected. As shown in the last row of Table 1, critical exponents for the ^PPIN^DNBs and ^TRN^DNBs subnetworks are significantly smaller (*p* < 0.0001 in both tests) than values estimated for the entire networks, thus supporting the above conclusion of DNBs being a class of particularly highly connected elements.

### 3.5. Mutations in Different Viral Proteins and Their Effect on the Likelihood of Disease Progression

The collection of TEV genotypes used in this study was limited to mutants in three viral proteins: HC-Pro, NIb, and CI. The strongest fitness effects were associated to mutations in the multifunctional HC-Pro (Figure 2). Indeed, these mutations were located in the RNA-binding domain of the protein and show a strong reduction in activity as suppressors of RNA silencing [32,76]. Plant immune systems easily control the spread of these viruses, resulting in mild symptoms and recovery from infection. Transcriptomic perturbations were small and genes experiencing a sharp transition in expression were mainly involved in pathogen detection. These mutant genotypes support the tenant of the DNB theory that the predisease state is reversible, as plants infected with low fitness mutants recovered from infection.

The only mutant we studied affecting the replicase NIb had a moderate fitness effect, but induced more severe symptoms and a major reorganization in the host transcriptome (Figure 2). Indeed, the DNB analyses have identified the disease category IV, induced by TEV NIb/PC95, as the tipping point for the irreversible transition from predisease to disease states. Such tipping points are network modules involved in response to infection, plant senescence and developmental defects, RNA and protein processing and trafficking, and reorganization of cellular components, especially organelles. Together all these alterations explain the triggering of stronger symptoms that can be seen in the sudden phase transition to the new equilibrium characteristic of the disease state.

Finally, we studied two mutations in the CI helicase protein, which had small deleterious fitness effects and were quite similar to the WT virus in their phenotypes (symptoms and transcriptomic perturbations). These viruses are highly virulent, and bring the plant to the most severe disease categories, already corresponding to the irreversible disease state in which the plant is strongly affected.

## 4. Conclusions

DNB theory has been successfully applied to predict tipping points of progression in human diseases such as hepatitis C virus-induced dysplasia and hepatocellular carcinoma [12,19], lung injury after carbonyl chloride inhalation exposure [12,19], and type 1 diabetes [77]. Since complex diseases result from the perturbation of the homeostatic functioning of multiple regulatory networks, the classic approach of using single genes to predict the evolution of a disease is, at best, of dubious utility. Instead, predictions should be based on the identification of leading network biomarkers: groups of genes that enter into resonance as the system gets closer to the irreversible transition from the predisease to the disease states. Here, we have used for the first time the mathematical formalism of the DNB theory to learn more about the progression of an infectious disease in plants. We have been able to define discrete states of tobacco etch disease progression by using mutant genotypes of the causing agent, TEV, which differ in their capacity to induce infections of varying severity that correlate with the magnitude of the perturbations in the plant transcriptome. Eight dpi, infected plants may have been capable of controlling disease and continue growing, may be still struggling with the virus as they approach to the critical phase transition point, or may well be beyond it in an irreversible disease state.

As predicted by the DNB theory, we found network biomarkers that underwent a sudden change in their dynamical behavior as they got closer to the phase transition: they showed strong correlations in their gene expression and an increase in variance and a loss of coordination with genes outside the DNB. We identified such DNBs in the context of both PPIN and TRN. The functional annotation of these DNBs illustrates classic mechanisms of host–virus interaction (e.g., activation of defense pathways, overactivation of secondary metabolism, chloroplast malfunctioning, etc.), but also highlights the role of genes not described before in relation with disease. Interestingly from a network’s perspective, we found that genes belonging to the DNB subnetworks were more central and highly connected than expected by chance. This is in good agreement with previous reports showing that host DEGs responding to virus infection tend to be hubs [73,74].

Our study has several obvious drawbacks. First, our results are entirely computational and should be taken at their face value: as suggestions for future research venues. The infection of plants carrying mutations in genes in the DNBs, along with the analyses of their transcriptomes and placing results in the context of PPIN and TRN, will confirm the value of our predictions. Second, the experimental data were generated for the TEV-tobacco pathosystem. However, given the lack of detailed information on tobacco PPIN and TRN and incomplete genome annotation, we had to rely on identifying *Arabidopsis* orthologues and on PPIN and TRN models inferred for this plant. By doing this mapping, we are obviously missing information that may be relevant to better understanding the infection process in tobacco. Finally, we report results from a single pathosystem. Whether our findings can be extrapolated to other plant pathosystems is a really open question. More viruses, especially sampling from a diverse array of life history strategies (e.g., persistent vs. acute infections), and host species need to be explored before making strong conclusions about the usability of this computational approach.

As a final remark, the use of new computational approaches to analyze and integrate omic data into the context of interaction and regulatory networks is becoming more common in biomedical research, allowing the identification of targets for new drugs, which instead of affecting the expression of individual genes would exert their effect throughout the properties of networks. Expanding this approach to plant health will clearly allow to identify new venues to create the next generation of pathogen-resistant plants.

## Figures and Tables

**Table 1:** Quantitative parameters describing the topology of global networks and DNB subnetworks. Values are reported as averages ±1 SD.

**Network Property**	**PPIN**	**^PPIN^DNB**	**TRN**	**^TRN^DNB ^1^**
Shortest path	3.529 ± 0.739	3.650 ± 0.990 *	3.476 ± 0.274	3.355 ± 0.216 *
Betweenness centrality	0.023 ± 0.388	0.015 ± 0.104	1.144 ± 3.739 × 10^−^^4^	2.612 ± 7.300 × 10^−^^4^
Closeness centrality	0.292 ± 0.094	0.308 ± 0.158	0.289 ± 0.022	0.299 ± 0.019 *
Clustering coefficient	0.138 ± 0.198	0.152 ± 0.242	0.213 ± 0.137	0.184 ± 0.107 *
Degree	20.138 ± 31.148	20.523 ± 41.789	58.905 ± 60.034	90.513 ± 85.910 *
Eccentricity	7.544 ± 1.300	7.530 ± 1.859	120.526 ± 82.141	5.930 ± 0.443 *
Neighborhood connectivity	65.840 ± 73.970	38.605 ± 45.927 *	58.898 ± 60.027	98.598 ± 53.418 *
Topological coefficient	0.161 ± 0.178	0.195 ± 0.222	0.096 ± 0.083	0.065 ± 0.050 *
Critical exponent degree distribution ^2^	−2.749 ± 0.399	−2.043 ± 0.366 *	−3.225 ± 0.174	−2.512 ± 0.210 *

^1^ Without including ^TRN^DNB-1 in the computations. ^2^ Calculated from the fit of the degree distribution to a power law probability density function. * Statistically significant difference between the entire network and the DNBs subnetwork (2 samples *t*-test).

## Supplementary Materials

The following are available online at https://www.mdpi.com/1999-4915/12/1/16/s1, Table S1: List of genes showing a significant biphasic behavior in gene expression. Table S2: List of enriched functional categories among genes listed in Supplementary Table S1. File S1: Computations of *I** and list of genes for each PPIN-based DNB (^PPIN^DNB). File S2: Computation of *I** and list of genes for each TRN-based DNB (^TRN^DNB).
